# Parents’ Sharenting Behaviours: A Systematic Review of Motivations, Attitudes, Perceptions, and Impression Management Perspectives

**DOI:** 10.12688/f1000research.161540.1

**Published:** 2025-04-17

**Authors:** Saeid Motevalli, Rogayah A. Razak, Richard Peter Bailey, Amalia B. Madihie, Katayoun Mehdinezhadnouri, Yifei Pan

**Affiliations:** 1 Psychology, UCSI University, Kuala Lumpur, Federal Territory of Kuala Lumpur, 56000, Malaysia; 2 Education, UCSI University, Kuala Lumpur, Federal Territory of Kuala Lumpur, 56000, Malaysia; 3 Counselling Programme, Universiti Malaysia Sarawak, Kota Samarahan, Sarawak, 94300, Malaysia

**Keywords:** Sharenting Behaviour, Parental Motivations, Digital Parenting, Good Health and Well-being, Motivation, Attitude

## Abstract

**Introduction:**

Sharenting involves parents sharing photos, videos, or other information about their children on their social media profiles via online platforms. Research indicated the rising prevalence of parental sharenting behaviour among various countries.

**Objective:**

The main aim of this article was to explore the role of motivations, perceptions, attitudes, and impression management on parental sharenting behaviours.

**Methods:**

A systematic review examined empirical studies published from 2019 to 2024 regarding parental motivations, attitudes, perceptions, and impression management associated with sharenting. Relevant studies were identified via Scopus and manual reference searches, with data extraction concentrating on study characteristics, demographics, objectives, design, and principal findings.

**Findings:**

Parental sharenting is motivated by intrinsic desires, social validation, and impression management, as parents curate content to improve their social image. While children value favourable representations, many object to sharing without consent. Notwithstanding privacy concerns, parents frequently prioritise advantages, raising ethical enquiries regarding children’s autonomy, privacy, and digital identity in digital self-representation.

**Conclusion:**

Parental sharenting, motivated by emotional satisfaction, social validation, and impression management, frequently neglects privacy risks and ethical considerations. Such practices may compromise children’s autonomy, privacy, and digital identity, resulting in conflicts with their rights. Children’s varied responses underscore these dilemmas, highlighting the necessity of reconciling parental intentions with safeguarding children’s digital futures and overall well-being.

**Recommendation:**

Parents should engage in mindful sharenting, policymakers must safeguard children’s digital rights, professionals should enhance awareness, and researchers should investigate methods to reconcile parental desires with children’s welfare.

## 1.0 Introduction

Sharenting, becoming more prevalent, involves parents sharing photos, videos, or other information about their children on their social media profiles via online platforms. ^23^ Research indicates that 74% of parents are acquainted with at least one other parent who overshares information regarding their children on social media ^28^ and that 10% of parents share details about their children’s health concerns. ^49^ The rising trend may seem harmless for parents to document their children’s lives and share milestones with family and friends. Parents often share to express pride, inform family, and create digital archives. ^77, 6^ These motivations are frequently well-intentioned, as social media offers a convenient method of documenting and commemorating a child’s life while maintaining communication with loved ones.

Nevertheless, sharenting raises substantial concerns regarding children’s privacy, safety, and future digital identity despite these positive aspects. ^12, 6^ Some researchers argue that sharenting can be considered a form of child abuse and neglect. ^21^ Children may be exposed to a variety of risks as a result of this new phenomenon, such as identity theft, sexual exploitation, and possible emotional distress in the future due to the public sharing of their feelings and experiences. ^16^ Uhls and Greenfield ^70^ discovered that sharenting may influence children’s self-perceptions, familial relationships, and interpersonal interactions. Moreover, sharenting could affect a child’s sense of self and identity and their capacity to navigate the online environment. In this regard, sharenting emphasises the critical need for greater awareness and the development of clear guidelines concerning the online sharing of children’s personal information.

### Sharenting and motivation

Self-determination theory (SDT ^64^;) employs the concept of innate, universal, and psychological needs to comprehend human motivation. In the context of parental sharenting, this framework can shed light on why parents share their children’s lives on social media. Ryan and Deci ^65^ argued that intrinsic and well-internalised extrinsic motivation is enhanced by supporting basic psychological needs for autonomy, competence, and relatedness. Parents may be motivated by intrinsic factors, such as the desire to document their child’s milestones for personal fulfilment (relatedness) or to feel competent as parents when they receive validation from online communities. External motivations—like social approval—can also play a role if parents seek recognition or social status. Extrinsic motivation, driven by external rewards or constraints, can sometimes undermine intrinsic motivation. ^64^ Intrinsic motivation is more effective than extrinsic motivation. ^41^

Many parents share content to capture and preserve important moments in their children’s lives, resulting in a digital archive that acts as a record for themselves and a tool to share these memories with family and friends. According to Kumar and Schoenebeck, ^40^ parents of young children typically share four types of pictures of their children on social networking sites: pictures of milestones (e.g., first teeth pictures), pictures with family and friends (e.g., holidays), and pictures they consider to be cute (e.g., baby pictures) or funny (e.g., eating dirt). Similar findings can be found in the research conducted by Brosch, ^10^ which indicates that parents of young children primarily share photographs of joyful occasions. These photographs include everyday life (for example, mealtimes), excursions (for example, holidays), and special events (for example, birthday parties).

In addition to being a location where parents post about their children, social media provides a platform for parents to discuss their parenting experiences and get parental assistance. Based on an interview survey conducted by Latipah et al., ^42^ parents’ motivation for sharenting is to receive social support and recognition from the community. According to Wagner and Gasche, ^75^ some mothers contribute to validate their parenting, to advise others, or to establish a network, among other reasons. Moreover, Lazard et al. ^46^ reckon that parents also utilise social media platforms to demonstrate their effective parenting. It is common for parents to share their experiences on social media platforms, such as when their children are diagnosed with a chronic illness. ^36^ In this case, they would post updates on social media regarding how they address these issues and remind other parents of the potential consequences.

Economic incentives may also be a factor, particularly for parents who engage in the influencer culture. Sharing family-related content can result in sponsorships, brand deals, and other financial benefits. Researchers indicated that the number of followers parents have on their social media platforms is a catalyst for sharing content related to children. ^10, 57^ Children can participate in promotional activities for brands, provided that their parents have an online identity that can be monetised, such as professional influencers. ^1, 20^ Celebrity’s parents and influencers frequently promote specific brands in their posts by incorporating them into their daily lives, thereby fostering a sense of intimacy and connection with their followers. ^11^ In addition, a somewhat distinct type of celebrity performance, known as “micro-celebrity parental mediation,” has also been the subject of Leaver’s research. ^45^ Parents post content about their children on a different "child’s own" profile to make money.

### Sharenting and attitudes

Parents’ attitudes toward sharenting are intricate and frequently influenced by positive intentions and underlying concerns. Parents frequently share pictures and stories about their children to express affection and pride in their accomplishments. In return, they obtain support and encouragement from family members and friends within their familial network, which alleviates stressful conditions, cultivates a sense of security, ^11^ and aids in the development and maintenance of social connections. ^23^ Sharenting was found to be linked with a variety of positive results, such as the normalisation of male parenting through the sharing of personal experiences of parenthood or the assistance of other parents in enhancing their parenting experience. ^2^

As digital connectivity continues to expand, there is an increasing demand for education and awareness regarding the consequences of sharenting for both parents and children. ^24^ Parents who share their children’s multimedia content online for a variety of psychosocial reasons leave themselves open to criticism from social network users. Besides, sharenting can also lead to privacy risks, potential misuse of children’s information, and conflicts with family members. ^33, 24, 56^ Brosch ^10^ pointed out that digital kidnapping is also a trend that occurs on social media when an individual misappropriates the child’s photographs and videos across all websites.

For this matter, some parents deliberately avoid sharing information about their child; when they do, they use strategies to reduce the risks of sharing. According to Autenrieth, ^3^ this is known as “anti-sharenting,” which refers to particular behaviours that parents use to obscure their child’s identity when posting images of them online. Parents have created new photo practices to balance the need to post images of their kids online with the desire to leave as few visual traces as possible. In this way, some parents can ensure that their child’s privacy is not violated by modifying the photographs, while at the same time being able to take advantage of the advantages that come with sharenting. ^75^ In addition to the method of photographing and editing the image, it is crucial for certain parents to prevent the inclusion of any additional identifying information, ^76^ such as using the child’s initials or pseudonyms, adjusting social media post reach, and sharing stories with specific followers through Facebook and Instagram private groups, as well as only posting images via Messenger or WhatsApp.

### Sharenting and perceptions

The social and psychological advantages parents derive from sharing their children’s lives online are frequently associated with the perceptions of sharenting as a form of validation. For parents, sharenting is a platform to exchange advice regarding parenting challenges and seek affirmation and support. ^49^ It allows parents to interact with their online networks and seek validation, support, and acknowledgement, which can boost their parental pride. Similarly, Duggan et al. ^22^ pointed out that sharenting can alleviate the sense of isolation parents experience when encountering challenges and inquiries during the parenting process as they seek guidance from their online community. Moreover, parents receive emotional support and establish connections with individuals who share their values through sharenting. ^10^

It is important to mention that research indicates that parents who are more aware of privacy concerns and have a higher level of digital literacy are more likely to participate in sharenting. ^53^ These parents frequently engage in sharenting while simultaneously implementing strategies to mitigate privacy threats, despite their comprehension of the potential risks of sharing their children’s personal information online. Some parents practise “mindful parenting,” which involves maintaining a balance between safeguarding their child’s privacy and sharing significant family events. This method entails the deliberate sharing of content, utilising privacy settings, and managing the quantity and type of information disclosed. ^76^ Furthermore, celebrity parents frequently disclose their children’s lives as part of their overall personal branding, which can result in lucrative sponsorships or media exposure. ^62^ This type of sharenting, which is influenced by both personal and financial incentives, emphasises the ambiguity between private family moments and public content intended for mass consumption.

Parents frequently share information out of pride or to update family and friends. ^77^ However, this practice raises significant concerns regarding children’s privacy and safety. ^12, 43^ The ethical implications of utilising children’s personal experiences to seek social acceptance and the possibility of oversharing have raised serious questions about the validation parents gain from sharing online. Moreover, there are digital footprints left behind by the information that parents share about their children, ^67^ and this information unconsciously affects the construction of an online identity for their child. ^49^ Nonetheless, this online representation may be in opposition to the manner in which adolescents endeavour to present themselves. When children are younger, not yet engaged with social media, lack formed opinions, or are too immature to articulate their views regarding their parents’ sharenting practices, it may be simpler for parents to disregard their perspectives. ^32^ Thus, critics argued that using the child’s image and experiences to gain social approval can violate the child’s privacy and autonomy.

### Sharenting and impression management

Impression management, also referred to as self-presentation, is the process by which individuals endeavour to regulate their own perceptions of themselves. ^27^ In the context of sharenting, impression management is a critical factor, as parents meticulously select the content they share about their children to influence how their social networks perceive them. In numerous instances, sharenting is transformed into a form of performative impression management, in which the content shared is not solely intended to document a child’s life but also to create a desired image of parenthood. Parents may intentionally share moments that reflect positively on their parenting abilities while avoiding content that could potentially portray them in a less favourable light.

Impression management is primarily influenced by an individual’s aspirations regarding their desired self-presentation and the societal expectations associated with their social roles. ^44^ Subsequently, individuals endeavour to portray themselves as proficient in their respective roles. ^44^ Parents aim to demonstrate their parental competencies by sharing information about family activities and how they address educational challenges. ^77^ Similarly, researchers indicated that parents aspire to be regarded as exemplary caregivers and demonstrate their parental abilities by disseminating specific content regarding their children. ^15, 40^ Sharenting, therefore can be considered a kind of impression management on their parenting performance. ^15, 40^ For "mommy bloggers" and social media influencers, impression management serves as a tool for developing a personal brand in addition to being about how one presents oneself. In such situations, sharing is often linked to money-making goals since sharing a picture of a perfect or ideal family life can get more followers, sponsors, and endorsements. ^62^

Scholars indicated that through sharenting, parents construct family narratives and identities while fostering connections with extended family members who interact with the posts. ^18^ Research from Collett ^15^ investigated how the mother is reflected in portraying children (e.g., their clothes) through participant observations. According to the findings, mothers genuinely use their children to validate their motherhood and to project a positive image of themselves, which raises their subjective well-being. ^15^ Bartholomew et al. ^5^ discovered that 93% of parents expect acknowledgement when sharing photos of their children, with both mothers and fathers indicating increased parenting satisfaction when friends provide feedback on these photos. However, concerning the perspective of children on impression management, Ouvrein and Verswijvel ^58^ found that parents’ sharenting behaviour distorts the children’s digital image, interferes with their digital self-representations, and disrupts their impression management efforts, despite the fact that children invest a significant amount of time and effort into constructing intentional online images and identities.

## 2.0 Research Question

1. What is the role of intrinsic and extrinsic motivations on parental sharenting behaviours?
2. What are the dominant perceptions of parental sharenting behaviour?
3. What are the positive and negative attitudes parents and children have towards sharenting?
4. What role does impression management play in influencing parents’ sharenting behaviour?

## 3.0 Method

### Eligibility criteria

Studies were selected if they met the following criteria: published in English and full-text was available; published between 2019 and 2024. Studies were eligible if they examined parents’ motivations, attitudes, perceptions, or impression management behaviours related to sharenting, defined as the practice of parents sharing information, images, or videos of their children on social media. Eligible populations included parents with at least one child across diverse cultural backgrounds. Articles were required to report empirical data on at least one of the four main aspects of sharenting behaviour: motivations for sharenting, attitudes toward the practice, perceptions among parents or the public, or impression management strategies in the context of parental sharing online. Studies employing qualitative, quantitative, or mixed-method designs were included, while review articles, commentaries, and case reports were excluded to maintain an emphasis on primary research.

### Search strategy

A systematic and comprehensive search was conducted to identify relevant studies. A literature search was performed on Scopus on 20th October 2024. The search strategy involved using multiple combinations of keywords, including “sharenting”, “motivation”, “perception”, “attitude” and “impression management”, which were combined using Boolean operators (e.g., AND, OR). The search was limited to studies published between 2019 and 2024. Additional manual searches were performed by reviewing the references to the articles included. Studies obtained from the search were transferred into the Excel database, and duplicates were removed. We searched reference lists and carried out citations, searching for included papers and previous reviews in this area.

### Data extraction

One reviewer conducted the literature search and extracted data into an Excel database. Five reviewers conducted an independent screening of titles and abstracts to assess their eligibility, thereby reducing the potential for selection bias. Each study was evaluated according to established inclusion criteria, which encompassed relevance to the research question, study design, and the availability of essential variables, including study population, sex, mean age, and sampling strategy. At this stage, studies that evidently did not fulfill the established criteria were excluded. To maintain consistency, any discrepancies among reviewers were addressed through discussion. Subsequently, a comprehensive review of the full texts of the remaining studies was undertaken to extract detailed information and ascertain their eligibility. The data extraction encompassed the following elements: author, year of publication, country of study, study population, gender, mean age, study design, research objectives, sampling methods, and principal findings.

To assess the quality of the studies included, a narrative evaluation of the risk of bias was performed, taking into account methodological rigor, sample selection, and potential biases in data collection and reporting. Research that depended on self-reported data was deemed to be at a significant risk of measurement bias, as participants may have offered responses that were socially desirable. Furthermore, research employing non-probability sampling techniques was evaluated as possessing a moderate to high risk of selection bias, thereby constraining the generalizability of the results. Some studies also lacked detailed methodological transparency, increasing the risk of reporting bias. No formal risk of bias assessment instruments, such as ROBINS-I or Cochrane RoB 2, were utilized; however, the limitations of the study were addressed through a narrative discussion to underscore potential biases.

The degree of certainty regarding the evidence was evaluated based on the study design, sample size, consistency of results, and the presence of potential biases. Owing to the heterogeneity of the studies included, the GRADE (Grading of Recommendations, Assessment, Development, and Evaluations) methodology was not implemented. Nonetheless, findings derived from studies employing larger sample sizes and rigorous methodologies were deemed to possess a higher degree of confidence, whereas studies characterized by smaller sample sizes, qualitative methodologies, or subjective measures were considered to exhibit lower certainty.

Because of the variation in study designs, populations, and measurement instruments, a narrative synthesis approach was used to examine and combine the results from the included studies. Because of these differences, a structured method was employed to classify and interpret the data rather than performing a meta-analysis. Based on their research focus, the studies were first categorized into thematic groups, such as impression management practices, parental motivations for sharenting, and attitudes of both parents and children. o find reoccurring patterns pertaining to parental decision-making, social validation, and ethical considerations, a thematic analysis was conducted for qualitative studies. To identify similarities and differences in the results, these themes were compared between studies. Descriptive statistics, correlation coefficients, and effect estimates were examined in quantitative research. However, no standardized effect measures were pooled because of differences in study methodologies. To sum up, a systematic tabular format was employed to delineate the essential characteristics of the included studies, including study design, demographic information, principal findings, and significant conclusions. The findings were subsequently synthesized narratively to emphasize consistencies, discrepancies, and areas necessitating additional investigation.

## 4.0 Result

### Study selection

**Figure 1.**
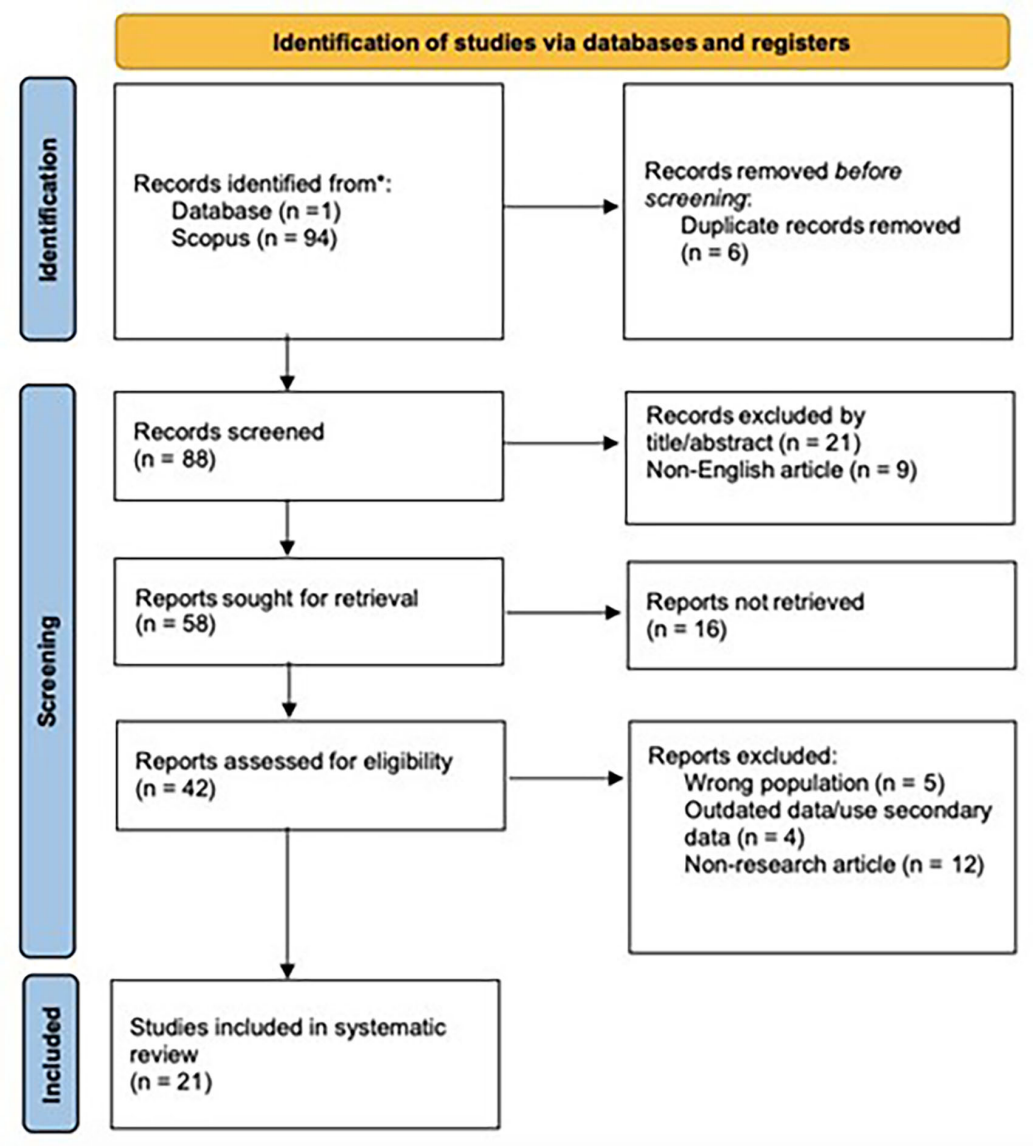
Study selection using the PRISMA flow diagram. *Note.* Adapted from Page et al., 2021.

21 articles were obtained from 1 database, as shown in Figure 1 and detailed in the appendix (Extended Data Table). After removing six duplicates, 88 articles remained. Subsequently, 21 articles were excluded following the screening of titles and abstracts, and 9 were excluded because they were not published in English. Eventually, 65 abstracts met the inclusion criteria.

### Characteristics of included studies

The studies included in this review were published between 2019 and 2024. This systematic review synthesizes the findings from 21 studies that examine parental sharenting behaviors, the motivations underlying these behaviors, privacy concerns, as well as their psychological and social ramifications. The studies encompassed in this compilation originate from thirteen countries, namely Belgium, Spain, the United Kingdom, the United States, Turkey, Portugal, Indonesia, Malaysia, China, Estonia, the Czech Republic, Venezuela, and Nepal. These studies utilize a diverse array of research methodologies, comprising ten qualitative studies, four cross-sectional studies, one mixed-methods study, and one randomized controlled trial. The total sample size varies across studies, ranging from 8 to 2900 participants, with most studies using non-probability sampling. The ages of participants exhibit considerable variability, with mean ages documented between 15.5 years for adolescents and 61.06 years for grandparents, thereby illustrating a broad spectrum of demographic viewpoints regarding the phenomenon of sharenting.

### Sociodemographic and cultural disparities in sharenting

Parental sharenting behaviors are influenced by factors such as age, gender, educational attainment, and cultural norms. Numerous studies indicate that younger parents, particularly mothers, are more likely to participate in sharenting with greater frequency. ^33, 55, 38^ Furthermore, research derived from cross-cultural comparisons reveals that parents in Western nations, such as Belgium, the United Kingdom, and the United States, place a premium on self-expression and peer interaction. In contrast, parents from non-Western countries, including China, Malaysia, and Indonesia, tend to prioritize familial connectivity and the documentation of experiences. ^39, 80, 69^ Research examining social media platforms indicates that Instagram and Facebook are the predominant channels utilized for sharenting, while TikTok is regarded as a high-risk platform owing to its open-access characteristics. ^73^

### Motivations on sharenting behaviours

Numerous research identifies intrinsic and extrinsic motivations as influential factors in parents’ engagement with sharenting behaviours. Intrinsic motivations include emotional satisfaction, which was found in six of the included articles, with motivations ranging from documenting memories ^69, 33, 42, 67, 80^ to maintaining emotional connections with family and friends. ^80, 77, 33^ Pride in children’s achievements was also an intrinsic motivation reported in four articles, including motivations to share milestones. ^77, 33, 67, 80^

Extrinsic motivations included seeking social validation, which was mentioned in one article, ranging from demonstrating good parenting skills ^42^ to engaging with online communities. Peer influence and social norms were noted in five articles, with motivations including conforming to societal expectations ^55^ and the influence of supportive networks. ^80, 42, 63, 67^ Impression management was identified in three articles, with motivations to portray oneself as a caring and attentive parent. ^67, 42, 55^ These motivations generally depict a complex interplay between emotional needs, social pressures, and self-presentation goals that drive parents to engage in sharenting behaviours.

**Table 1.** Intrinsic and Extrinsic Motivations on Sharenting Behaviours.

Researcher(s)	Year	Perspective	Intrinsic Motivation on Sharenting	Extrinsic Motivation on Sharenting	Significant Information
Ranzini et al.	2020	Parents		A strongly supporting offline network, such as close friends or family members, positively relates to the frequency of sharenting	
Latipah et al.	2020	Parents	For documentation	- To receive affirmations and social support - To show the ability to care for children - For social participation	
Hinojo-Lucena et al.	2020		- Sharing family moments - The picture is hilarious - Intention to keep that memory online - A desire to make the child known - Showing off for contacts		
Ögel-Balaban	2021	Parents		- Present valued intimate relationships online to create a positive image of themselves and their children - Probably following the cultural norms and expectations	
Tan & Dhanapal	2022	Parents	It will be easier for them to track their memories from social media apps in the future		They shared and posted a lot on social media, not to attract people to follow, subscribe to, or give likes and comments to them but purely to keep the memories online and be able to view them again.
Walrave et al.	2022	Parents	- Proud of them - Wanted to inform their family and friends		
Staes et al.	2023	Grandparents	- Save their most treasured memories - Inform others about the development of their grandchild - Proud of their offspring and happy to be their grandparent	- Interactions with other grandparents - Advise other grandparents about getaways or activities they can do with their grandchildren - Confirm the social role participants take on as a grandparent	
Zhu et al.	2024	Mothers	- Almost every mother in this research stated she uses SNS mainly to feature her child’s milestones, achievements and special moments - Mothers use sharenting to update family and close friends regarding their children’s life and development	- Getting emotional support or informational help	

### Perceptions of sharenting among parents

The reviewed studies present a comprehensive overview of parents’ diverse perceptions of sharenting. Privacy concerns were noted in two articles, where parents expressed concerns about the risks of sharenting, particularly regarding privacy and the potential misuse of their children’s information. ^9, 61^ However, three articles indicated that while parents have privacy concerns related to sharenting, their perception of the associated risks is often diminished by cognitive biases or unconscious factors. ^47, 2, 4, 51^ One article discussed the commercialisation of sharenting, particularly in the context of influencers who use their children’s images for financial gain. ^73^ Despite these concerns, many parents continue to share photos and information about their children on social media, frequently underestimating the potential risks to their children’s privacy and identity. To sum up, it suggests that while privacy and ethical concerns are prevalent, many parents’ perceptions of the risks involved in sharenting are often outweighed by their desire to share and celebrate their children’s lives online.

**Table 2.** Positive Perception, Neutral, and Negative Perception of Sharenting Behaviours.

Researcher(s)	Year	Perspective	Positive Perception	Neutral	Negative Perception
Lipu & Siibak	2019	Mothers	Most mothers felt comfortable sharing photos and information about their children on SNS. They were not very concerned about the potential of introducing new risks to their children’s identities and privacy.		
Briazu et al.	2021	Mothers			Concerns focused on identifying a child’s location and were often linked to safeguarding issues
Barnes & Potter	2021	Parents	Despite many respondents viewing this behaviour as risky, sharenting is a widespread practice.		
Amon et al.	2022	Parents	Parents who shared more frequently did not acknowledge a heightened risk of their child’s photos being used and manipulated by others, suggesting this is not a major concern for them.		
Peng	2023	Parents		Most participants are sensitive to information that exclusively belongs to their children and are aware of the potential risks of posting children’s information online without privacy protection.	
Vizcaíno-Verdú et al.	2023	Parents	Parents share the view that one of the reasons why influencers engage in sharenting is for promotional purposes.		

### Attitudes toward sharenting

Among the reviewed studies, a few studies highlighted positive attitudes toward sharenting. Two articles emphasised its positive role in maintaining family connections and sharing milestones, ^2^ as well as engaging in sharing for affirmation, support, and joyful moments, feeling compelled to post photos to reap these benefits. ^76^ Two articles indicated that children appreciate sharenting when it portrays them positively or celebrates their achievements, ^77^ fostering a sense of pride and connection with their parents. ^47^ However, one article highlighted that children express discomfort when their parents share content without their consent, particularly when it includes personal or embarrassing information. ^47^ In conclusion, it reveals that while sharenting is often perceived positively by both parents and children for fostering connections and celebrating achievements, significant concerns about privacy and lack of consent, especially from children, highlight the need for a more cautious approach.

**Table 3.** Positive and Negative Attitudes Toward Sharenting Behaviours.

Researcher(s)	Year	Perspective	Positive Attitudes	Negative Attitudes	Significant Information
Lipu & Siibak	2019	Pre-teens	The pre-teens were happy and proud when they noticed their mothers were sharing posts about their achievements or had posted photos reflecting their happy family life.	Children did not want their parents to share unflattering visuals (e.g. ‘ugly photos’ or ‘when my hair is messed up’), which would reflect negatively on their self-images.	Struggles between the parents and the children might occur because children’s privacy expectations were violated and not taken seriously by the parents
Amon et al.	2022	Parents	Parents are comfortable posting photos of their children online, relatively comfortable with friends and family sharing photos of their children, and rarely object to others’ parental sharing practices.		
Walrave et al.	2022	Adolescents	Almost all adolescents were positive toward sharing information about family activities or vacations. The adolescents perceived those posts as ‘nice and cute’, as long as the adolescents looked good in the pictures.		
Walrave et al.	2023	Parents	Some parents need to engage in sharenting for affirmation, support, and sharing joyful moments. So, these parents also feel a need to post photos to experience these benefits.		

### Impression management in sharenting

Among the reviewed studies, four articles discussed the role of impression management in sharenting. As reported by one article, parents engage in sharenting to portray themselves as caring and involved, which helps to enhance their social standing and parental identity. ^67^ Three articles highlighted that parents consciously curate the content they share, selecting positive moments that emphasise their roles as supportive and involved caregivers. ^55, 67, 80^ The motivation to present a favourable image on social media often leads to selective sharing, where parents avoid posting negative or challenging moments of parenting. In summary, impression management is a significant factor influencing sharenting behaviours, with parents using social media platforms to shape how others perceive them, both within their social circles and the broader online community.

**Table 4.** Role of Impression Management of Sharenting Behaviours.

Researcher(s)	Year	Perspective	Role of Impression Management	Significant Information
Staes et al.	2023	Grandparents	- Confirming their role online even when offline, the role of the grandparent was limited - Show off with their grandchildren to their online networks and mainly, the successes of their grandchildren were shared on social media, although they did not reflect reality	
Zhu et al.	2024	Mothers	Mothers do intentionally screen out negative information about their children.	Mothers believe they use social media to construct an authentic identity for themselves and their children.
Ögel-Balaban	2021	Parents	Engaging in sharenting to represent themselves, their children, and their families in a positive way	

The results of this review suggest a possible impact of reporting biases, particularly publication bias and selective outcome reporting. Most included studies were published in English-language journals, raising concerns about linguistic bias that may have led to the exclusion of relevant research from non-English sources. Moreover, the preponderance of studies originating from Western contexts indicates a geographical publication bias, thereby constraining the applicability of findings across diverse cultural settings. Furthermore, certain studies exhibited selective reporting of outcomes, wherein researchers highlighted the favorable aspects of sharenting behaviors while offering minimal discourse on the associated risks and ethical considerations. A limited number of studies provided effect sizes, confidence intervals, or comprehensive statistical results, thereby complicating systematic comparisons of the findings. The absence of pre-registered protocols for numerous studies included in the analysis exacerbates the likelihood of selective outcome reporting.

The overall certainty of evidence in this systematic review exhibited considerable variability among the studies. Research employing larger, well-defined sample sizes, validated measurement instruments, and rigorous methodologies has exhibited greater reliability in findings pertaining to parental motivations, privacy concerns, and impression management behaviors. Nevertheless, research that depends on self-reported data, employs cross-sectional designs, and utilizes convenience sampling is correlated with diminished confidence in the findings, owing to the potential for recall bias, social desirability effects, and inherent limitations in sampling. An additional challenge in evaluating certainty was the inconsistency in measurement instruments and definitions of outcomes across various studies. The absence of standardized instruments for assessing parental attitudes and children’s reactions to sharenting has led to variability in reporting, thereby complicating the consistent synthesis of evidence. While the findings across various studies converge in recognizing significant themes, including the influence of social validation and concerns regarding privacy, the lack of longitudinal studies and experimental research designs constrains the capacity to establish robust causal inferences.

## 5.0 Discussion

This systematic review suggests that intrinsic and extrinsic motivations influence sharenting behaviours. Although there is some awareness of privacy concerns, parents often underestimate these risks, which leads to frequent sharing. Some children appreciate positive portrayals, while others experience discomfort, particularly when content is shared without consent. Besides, sharenting behaviours are significantly influenced by impression management, as parents selectively share favourable moments to enhance their social image and reinforce their parental identity.

### What is the role of intrinsic and extrinsic motivations on parental sharenting behaviours?

In terms of the motivations for sharenting behaviour, it was found that parents’ sharenting behaviours are influenced by intrinsic and extrinsic motivations. Intrinsically, parents seek emotional satisfaction through documenting memories, ^69, 33, 42, 67^ contacting friends and families, ^33, 77, 67^ as well as celebrating their children’s achievements. ^77, 33, 67, 80^ It was consistent with the previous research. ^8, 72, 29, 54^ Extrinsically, parents are motivated by societal norms, ^55^ and supportive networks. ^63, 42, 67, 80^ Many seek approval from social networks and align with expectations of "good" parenting by curating content that portrays them as attentive caregivers. ^42, 55, 67^ It aligns with the previous research. ^72, 53, 31^

Building on the intrinsic and extrinsic motivations identified for sharenting, it is essential to consider the broader social and psychological influences that amplify these behaviours. For instance, online photo-sharing provides viewers with continuous opportunities to engage with images through Likes and comments, which parents may interpret as feedback validating their portrayal of themselves and their children. ^24^ A study found that when new mothers receive likes and positive comments in response to the photos and videos they share of their babies via social media, it validates them as good mothers and makes them feel supported. ^40^ As digital platforms increasingly facilitate instant feedback, parents may depend more on external affirmation, reinforcing sharenting behaviours. Research has shown that baby photos often receive a higher volume of Likes than other posts, ^40^ and posts mentioning a child’s name attract increased attention. ^52^ This pattern may subtly shift parental self-esteem, aligning it more closely with online validation than intrinsic fulfilment. Furthermore, the economic incentives associated with sharenting have expanded, prompting some parents to utilise their online profiles as platforms for brand-building. Individuals who embrace micro-celebrity as a profession attain fame and financial benefits through their children’s involvement. ^60^ In this regard, sharenting manifests in various ways, from celebrity parents prominently featuring their children on Instagram ^37^ to father influencers engaging in “sharenting labour” as a means of commercialising the concept of involved fatherhood. ^11^ However, as children’s identities are used to generate income without fully understanding the consequences, these economic pursuits raise ethical questions about autonomy and consent. For this matter, Beuckels and colleagues ^7^ discovered that parent influencers possess limited awareness of the risks linked to sharing content featuring their children online. Besides, researchers discovered that parents view influencer sharenting as morally deficient and as converting children into marketing tools. ^73^ Therefore, sharenting behaviour reflects a combination of both intrinsic and extrinsic motivations, along with the pressures and ethical complexities involved in navigating the personal, social, and financial domains in the digital age.

### What are the dominant perceptions of parental sharenting behaviour?

Regarding the perception of sharenting behaviour, despite acknowledging privacy concerns ^9, 61^), parents often underestimate the risks associated with sharenting. ^47, 2, 4, 78^ Some parents continue to share for financial gain or social connection, ^73^ giving celebrification more importance than the possible privacy consequences for their children. Sharenting has considerable implications; however, knowledge of its risks or previous adverse experiences did not result in parents refraining from posting their children on social media. ^9^ Similarly, Hoy and co-workers ^35^ discovered that although parents demonstrate caution in disclosing their own and their children’s information online, sharenting behaviour persisted. The preference for social and emotional advantages, like celebrification and connection, over the less concrete and more abstract effects of sharenting significantly contributes to this disparity. Nevertheless, parents who do not engage in this field of childhood celebrification advocate the protection of their children from a phenomenon that is shrouded in an aura of vernacular positivity. ^74^

Research by Vizcaíno-Verdú and colleagues ^73^ found that parents were generally aware of the risks of sharing their children’s content on influencer profiles, recognising that while the content appeared harmless, posting on platforms like YouTube, Instagram, and TikTok could affect their children’s futures. Indeed, parents neither criticise influencers nor frame minors as victims. ^43^ Rather, they underscore the importance of implementing measures to safeguard the well-being of children in a world where the line between physical and digital spaces is becoming more ambiguous. Recent evidence from Livingstone and co-workers ^48^ indicates that parents’ risk perception, among other factors, affects the strategies they employ to mediate their children’s online activities. In this way, some parents observed the potential of excluding their children from resilient celebrification on the internet or sharing photos of their children on private profiles that are only accessible to family and friends. ^45^ Moreover, research by Walrave and collaborators ^76^ on mindfulness sharenting indicates that parents recognise the potential advantages of sharenting and implement strategies to safeguard their child’s privacy while simultaneously reaping the benefits it provides them. These strategies encompass capturing the child from a distance, having the child avert their gaze from the camera, concentrating solely on a specific body part, obscuring the face with an emoticon, blurring the facial features, or cropping identifiable elements from the photograph. ^76^ In summary, parents’ perception of sharenting illustrate a conflict between acknowledging its dangers and appreciating its social and emotional advantages. While many persist in sharing, some exhibit increasing awareness by implementing strategies to alleviate privacy concerns, underscoring the evolving nature of parental attitudes towards digital sharing.

### What are the positive and negative attitudes parents and children have towards sharenting?

Concerning attitudes towards sharenting, parents regard it as a means to sustain connections and commemorate milestones. ^2, 76^ Nevertheless, children value favourable representations ^77^ but resent sharing without consent, particularly when it involves personal or embarrassing material. ^47^ It underscores the duality of parental and children’s perspectives on sharenting, weighing its perceived advantages against significant ethical dilemmas. Scholars pointed out that younger parents are more likely to use social networking sites (SNSs), feel more at ease there, and have a more positive attitude towards them. ^25, 30^ As a result, they may engage in sharenting more frequently.

Ouvrein and Verswijvel ^58^ discovered that the reactions to sharenting activities are not uniform. Children occasionally express gratitude for their parents’ posts due to their celebratory nature, demonstration of pride, and positive presentation. However, this is not invariably true, as parents disseminate images children perceive as embarrassing or flawed. This ultimately induces frustration as the parents’ sharenting practices undermine the children’s meticulously crafted digital identity. Similarly, research from Hiniker et al. ^32^ showed that adolescents frequently feel embarrassed by the content their parents share about them on SNSs and may experience frustration as a result. Moreover, adolescents with heightened concerns regarding online privacy are more inclined to disapprove of sharenting. ^72^ Furthermore, Sarkadi et al. ^66^ surveyed Swedish children, revealing their negative attitudes towards sharenting. The children expressed a desire to be consulted before photographs are taken and shared and to have their refusals acknowledged. This discomfort highlights a significant disparity in decision-making authority, wherein children’s perspectives are frequently omitted from conversations regarding their digital existence. Such practices provoke overarching enquiries regarding children’s autonomy, privacy, and the enduring consequences of their digital identities, which are progressively influenced by parental behaviours. In this regard, a recent qualitative study revealed that adolescents believe sharenting should be more carefully considered and limited when they recognise their representation in the online realm. ^58^ Besides, a few research indicates that children and adolescents prefer their parents to obtain consent before sharing about them on social media. ^58, 66^

### What role does impression management play in influencing parents’ sharenting behaviour?

Impression management has a significant impact on sharenting behaviours, with parents selectively sharing positive moments to portray themselves as caring and engaged carers, thereby improving their social image in both personal networks and the wider online community. ^55, 50, 67, 80^ Many studies have revealed that parents want others to view them as good parents and will thus exhibit their parental competences by distributing particular content about their children. ^15, 40^ Additionally, Crabtree and Pillow ^17^ found that the need to belong predicts an increased use of Facebook through strategic impression management.

As famously described by Goffman, ^27^ impression management is presenting oneself in an idealised way that aligns with desired perceptions. People curate their identity and self-esteem to get through social situations, earn rewards (likes), and stay out of trouble. ^44^ This performance has been analysed as an extended self, ^34^ as the delineation of self, ^8^ and as a form of representation. ^13^ According to Blum-Ross and Livingstone, ^8^ sharenting is a form of digital self-representation. Holiday et al. ^34^ further argued that the most notable motivation driving parental engagement in sharenting activities is the pursuit of self-representation, as parents seek to convey their identity through sharing content related to their children online.

Nevertheless, it raises ethical and developmental issues, as parental self-presentation may hinder children’s capacity to form their digital identities. It was contended that sharenting interferes with the digital self-representations of the children, distorts their digital image, and disrupts their impression management efforts. Ouvrein and Verswijvel ^58^ assert that sharenting may yield adverse effects due to its impact on children’s digital identity. This aligns with Davidson-Wall’s ^19^ findings that managing parental impressions regarding the child may conflict with the child’s developmental objective of establishing an autonomous identity. The sharenting behaviour of parents for impression management may inadvertently infringe on children’s privacy and self-representation rights, potentially restricting their future control over their digital identity and impacting social and psychological development.

## 5.0 Limitation

Nevertheless, it is imperative to acknowledge certain limitations. Primarily, numerous studies incorporated in this analysis depend on self-reported data from parents, which may be susceptible to social desirability bias. This bias could result in an inflated representation of favorable attitudes towards sharenting, while concurrently underreporting apprehensions regarding privacy infringements and ethical dilemmas. The geographic distribution of the studies constitutes a limitation, as the preponderance of research is derived from Western nations, with a comparatively scant number of investigations addressing non-Western contexts, where cultural norms pertaining to digital parenting may vary considerably.

Furthermore, the review process is subject to specific limitations. The search was performed utilizing Scopus and manual reference searches, which may have inadvertently omitted pertinent studies indexed in alternative databases, such as Web of Science or PubMed. The exclusion of publications in languages other than English significantly constrains the scope of the analysis, potentially overlooking critical findings from regions where sharenting practices and regulations may differ markedly. Furthermore, the synthesis of evidence predominantly depends on thematic analysis rather than meta-analysis, which constrains the capacity to quantify the strength of associations between sharenting behaviors and their psychological or social repercussions for children.

## 6.0 Conclusion

This systematic review indicates that sharenting is motivated by intrinsic factors, including emotional satisfaction and memory preservation, as well as extrinsic influences, such as social validation, financial incentives, and impression management. Parents frequently curate content to project an idealised parental identity, occasionally prioritising these motivations above the privacy and autonomy of their children. Despite recognising privacy risks, parents often undervalue the enduring implications of sharenting, which can jeopardise children’s digital identities and autonomy. The duality in children’s perspective—valuing positive representations while feeling uneasy about sharing without permission—highlights the ethical intricacies of this practice. Hence, sharenting embodies societal pressures to adhere to idealised parenting standards while utilising digital platforms for social and economic gain.

A collaborative initiative is required from parents, policymakers, professionals, and researchers to tackle these challenges. Parents should implement conscientious practices, such as securing their children’s consent, minimising identifiable information, and guaranteeing secure sharing methods. Moreover, policymakers must formulate comprehensive regulations to safeguard children’s digital rights and develop systems for addressing violations. Furthermore, professionals, such as educators and child advocates, ought to spearhead awareness initiatives that emphasise the ethical ramifications of sharenting and provide pragmatic advice for safer digital conduct. In addition, future research ought to examine methods to reconcile parental motivations with the rights and welfare of children, assess the enduring effects of sharenting on digital identity development, and formulate evidence-based strategies to alleviate privacy and ethical concerns. These comprehensive efforts can facilitate sharenting behaviour that honours both parental aspirations and children’s rights.

## Data Availability

No data are associated with this article. Fighshare: Parents’ Sharenting Behaviours: A Systematic Review of Motivations, Attitudes, Perceptions, and Impression Management Perspectives, DOI: https://doi.org/10.6084/m9.figshare.28341926.v3 ^81^ The project contains the following extended data:
• Extended Data Table. Characteristics of reviewed articles Data are available under the terms of the Creative Commons Attribution 4.0 International license (CC-BY 4.0). Fighshare: Parents’ Sharenting Behaviours: A Systematic Review of Motivations, Attitudes, Perceptions, and Impression Management Perspectives, DOI: https://doi.org/10.6084/m9.figshare.28341926.v3 ^81^ The project contains the following reporting guidelines files:
• PRISMA Checklist • PRISMA Flowchart Data are available under the terms of the Creative Commons Attribution 4.0 International license (CC-BY 4.0).
