# Supplementary material for: Parents’ Sharenting Behaviours: A Systematic Review of Motivations, Attitudes, Perceptions, and Impression Management Perspectives
Source: F1000Res. 2025 Jul 16;14:448. Originally published 2025 Apr 17. [Version 2] doi: 10.12688/f1000research.161540.2 (PMC12344405; doi:10.12688/f1000research.161540.2)
Supplement: Peer review history

# Reviewer report: Reviewer response for version 2

**DOI:** 10.5256/f1000research.182129.r398764

**Published:** 2025-08-12

**Authors:** Gergely Ferenc Lendvai

**Affiliations:** 1 Pázmány Péter Catholic University, Budapest, Hungary

Thank you for the authors for the revisions. I suggest indexing without reservations. This a great paper which, I think, will make a great impact in the field of sharenting research. Congratulations to the Authors!

Are the rationale for, and objectives of, the Systematic Review clearly stated?

Yes

Is the statistical analysis and its interpretation appropriate?

No

If this is a Living Systematic Review, is the ‘living’ method appropriate and is the search schedule clearly defined and justified? (‘Living Systematic Review’ or a variation of this term should be included in the title.)

No

Are sufficient details of the methods and analysis provided to allow replication by others?

Partly

Are the conclusions drawn adequately supported by the results presented in the review?

Yes

Reviewer Expertise:

QSS, law, digital rights

I confirm that I have read this submission and believe that I have an appropriate level of expertise to confirm that it is of an acceptable scientific standard.

# Reviewer report: Reviewer response for version 2

**DOI:** 10.5256/f1000research.182129.r398765

**Published:** 2025-07-25

**Authors:** Mohtaram Rabbani

**Affiliations:** 1 Hong Kong University, Hong Kong, China

I have reviewed the article, have no additional comments, and hereby approve it.

Are the rationale for, and objectives of, the Systematic Review clearly stated?

Yes

Is the statistical analysis and its interpretation appropriate?

Yes

If this is a Living Systematic Review, is the ‘living’ method appropriate and is the search schedule clearly defined and justified? (‘Living Systematic Review’ or a variation of this term should be included in the title.)

Yes

Are sufficient details of the methods and analysis provided to allow replication by others?

Yes

Are the conclusions drawn adequately supported by the results presented in the review?

Yes

Reviewer Expertise:

Parenting

I confirm that I have read this submission and believe that I have an appropriate level of expertise to confirm that it is of an acceptable scientific standard.

# Reviewer report: Reviewer response for version 1

**DOI:** 10.5256/f1000research.177580.r383741

**Published:** 2025-05-30

**Authors:** Gergely Ferenc Lendvai

**Affiliations:** 1 Pázmány Péter Catholic University, Budapest, Hungary

This is an interesting paper and I think it could be interesting once in publishable form. The authors clearly outline the research objectives and I think that given the topic's prevalence it could be a great addition to the literature. Nonetheless, there are multiple problems with the paper that I hope the authors will find time to consider.

 Introduction>

 Probably the most burning problem in the introduction is the lack of conceptualization. This is an issue as the reader only reads a very basic understanding of sharenting despite it being an extremely complex issue (cf. Sharenting as a regulatory paradox – a comprehensive overview of the conceptualization and regulation of sharenting | International Journal of Law, Policy and the Family | Oxford Academic, or Steinberg, 2017). I highly recommend introducing the concept in much more detail.

 I also miss the outlining of why this is a research gap given the fact that multiple studies have worked on this, e,g. Sharenting: A systematic review of the empirical literature - Tosuntaş - 2024 - Journal of Family Theory & Review - Wiley Online Library or From the Cradle to the Web: The Growth of “Sharenting”—A Scientometric Perspective - Cataldo - 2022 - Human Behavior and Emerging Technologies - Wiley Online Library. Given Tosuntas's highly similar work I think it is imperative to highlight what makes this study different. I would also recommend reading vd Abeele's work which is very similar in theme to this> Tell me why: The impact of mindful sharenting explanations by momfluencers: An experimental study with mothers - Elisabeth Van den Abeele, Liselot Hudders, Ini Vanwesenbeeck, 2025

 The structure of the introduction is also a bit puzzling as it is essentially a lit.rev. This is OK by me but a more rigorous peer reviewer would find it problematic so it might be worth considering a better segmentation. 

 The RQs are not reasoned. Usually, in studies like this, there should be a short explanation as to why these RQs were chosen, this is missing right now.

 METHODS

 The authors give no explanation as to why they chose a five year period. Furthermore, it is extremely problematic that they included 2024 while the data collection was done in October 2024. In systematic reviews or bibliometric works, this practice is highly undesirable as the authors miss out on other publications indexed in 2024. A much more rigorous explanation should be needed here to support this unorthodox choice.

 Furthermore, the PRISMA chart usually goes under the Methods not the results, but I leave it up to the authors to consider this recommendation.

 What I find a bit puzzling is that the authors specifically outline that they will include scientometric (quantitative) methods, e.g. DS, CC or estimates, however, they are nowhere to be seen in the paper. This is a grave mistake which is either to be corrected or removed wholly. Though the authors do mention that However, no standardized effect measures were pooled because of differences in study methodologies. a better explanation is needed because it is extremely confusing in the current MS.

 The other parts are adequate and all around OK. There are many awkward wordings, therefore, I would suggest reviewing the language one more time, sadly, the form does not allow me to highlight them one by one.

Are the rationale for, and objectives of, the Systematic Review clearly stated?

Yes

Is the statistical analysis and its interpretation appropriate?

No

If this is a Living Systematic Review, is the ‘living’ method appropriate and is the search schedule clearly defined and justified? (‘Living Systematic Review’ or a variation of this term should be included in the title.)

No

Are sufficient details of the methods and analysis provided to allow replication by others?

Partly

Are the conclusions drawn adequately supported by the results presented in the review?

Yes

Reviewer Expertise:

QSS, law, digital rights

I confirm that I have read this submission and believe that I have an appropriate level of expertise to confirm that it is of an acceptable scientific standard, however I have significant reservations, as outlined above.

# Reviewer report: Reviewer response for version 1

**DOI:** 10.5256/f1000research.177580.r379496

**Published:** 2025-05-20

**Authors:** Mohtaram Rabbani

**Affiliations:** 1 Hong Kong University, Hong Kong, China

This paper provides an in-depth exploration of the phenomenon of sharenting, examining various aspects such as motivations, attitudes, perceptions, and impression management. One of the paper's strengths lies in its comprehensive analysis, which covers a wide range of topics related to sharenting, including intrinsic and extrinsic motivations, privacy concerns, and impression management. This thorough approach offers a nuanced understanding of the topic and its implications.

 The paper also benefits from the inclusion of studies from diverse countries, employing various research methodologies. This diversity enriches the analysis by providing a broad cultural perspective on sharenting behaviors. Additionally, the integration of theoretical frameworks, such as self-determination theory and impression management theory, helps to frame the motivations and behaviors of parents in a structured manner, offering insights into the psychological underpinnings of sharenting.

 Furthermore, the paper critically examines the ethical concerns associated with sharenting, such as privacy violations and the impact on children's autonomy and digital identity. This critical perspective is essential for understanding the potential risks of sharenting. The paper concludes by offering practical recommendations for parents, policymakers, and researchers. It advocates for mindful practices and regulatory measures to address the challenges posed by sharenting.

 Despite its strengths, the paper could benefit from improvements in clarity and structure. Some sections might be more effectively communicated with more concise writing and clearer organization to enhance readability and flow. The reliance on self-reported data is acknowledged as a limitation, as such data may be subject to social desirability bias. Future research could incorporate more objective measures or observational studies to validate findings.

 The analysis predominantly focuses on Western contexts, which might not fully capture the diversity of sharenting practices globally. Including more studies from non-Western contexts would provide a more balanced view. Additionally, the mention of studies published between 2019 and 2024 suggests an inclusion of future dates, which should be corrected to reflect the actual timeframe of the research covered.

 While a narrative synthesis is provided, the absence of a meta-analysis limits the ability to quantify the strength of associations across studies. Including a meta-analysis where applicable could strengthen the statistical rigor of the findings. The discussion could also be enriched by exploring the role of digital literacy in shaping parents' sharenting behaviors and how education can mitigate risks.

 Overall, this paper offers valuable insights and recommendations on sharenting. By addressing the identified areas for improvement, it could further enhance its contribution to understanding the complexities of parental sharing behaviors in the digital age.

Are the rationale for, and objectives of, the Systematic Review clearly stated?

Yes

Is the statistical analysis and its interpretation appropriate?

Yes

If this is a Living Systematic Review, is the ‘living’ method appropriate and is the search schedule clearly defined and justified? (‘Living Systematic Review’ or a variation of this term should be included in the title.)

Yes

Are sufficient details of the methods and analysis provided to allow replication by others?

Yes

Are the conclusions drawn adequately supported by the results presented in the review?

Yes

Reviewer Expertise:

Parenting

I confirm that I have read this submission and believe that I have an appropriate level of expertise to confirm that it is of an acceptable scientific standard, however I have significant reservations, as outlined above.
